# An integrated proteo-transcriptomics approach reveals novel drug targets against multidrug resistant *Escherichia coli*

**DOI:** 10.3389/fmicb.2025.1531739

**Published:** 2025-02-25

**Authors:** Manisha Aswal, Nirpendra Singh, Neelja Singhal, Manish Kumar

**Affiliations:** ^1^Department of Biophysics, University of Delhi South Campus, New Delhi, India; ^2^Institute of Stem Cell Science and Regenerative Medicine, Bengaluru, India

**Keywords:** RNA-Seq, SWATH-LC MS/MS, protein–protein interactions, multidrug resistance, drug target

## Abstract

Infections due to multidrug-resistant (MDR) *Escherichia coli* are associated with severe morbidity and mortality, worldwide. Microbial drug resistance is a complex phenomenon which is conditioned by an interplay of several genomic, transcriptomic and proteomic factors. Here, we have conducted an integrated transcriptomics and proteomics analysis of MDR *E. coli* to identify genes which are differentially expressed at both mRNA and protein levels. Using RNA-Seq and SWATH-LC MS/MS it was discerned that 763 genes/proteins exhibited differential expression. Of these, 52 genes showed concordance in differential expression at both mRNA and protein levels with 41 genes exhibiting overexpression and 11 genes exhibiting under expression. Bioinformatic analysis using GO-terms, COG and KEGG functional annotations revealed that the concordantly overexpressed genes of MDR *E. coli* were involved primarily in biosynthesis of secondary metabolites, aminoacyl-tRNAs and ribosomes. Protein–protein interaction (PPI) network analysis of the concordantly overexpressed genes revealed 81 PPI networks and 10 hub proteins. The hub proteins (rpsI, aspS, valS, lysS, accC, topA, rpmG, rpsR, lysU, and spmB) were found to be involved in aminoacylation of tRNA and lysyl-tRNA and, translation. Further, it was discerned that three hub proteins - smpB, rpsR, and topA were non homologous to human proteins and were involved in several biological pathways directly and/or indirectly related to antibiotic stress. Also, absence of homology ensures a little cross-reactivity of their inhibitors/drugs with human proteins and undesirable side effects. Thus, these proteins might be explored as novel drug targets against both drug-resistant and -sensitive populations of *E. coli.*

## Introduction

*Escherichia coli* are common gut bacteria which are frequently reported from medical and veterinary clinics (Croxen et al., 2013; Nhung et al., 2015). The soaring use of antibiotics in agriculture, veterinary, poultry and clinics has increased the incidence of antimicrobial resistant bacteria, including *E. coli*. Several reports have indicated that *E. coli* have become resistant to many antibiotics of the beta-lactam, aminoglycoside, (fluoro) quinolone classes and also to the newer antibiotics like colistin, which is a matter of grave concern (Liu et al., 2016; Peng et al., 2021; Singh et al., 2022). Antimicrobial resistant infections have not only escalated the costs of medical treatment, in many cases it might lead to treatment failures (Dadgostar, 2019). According to an estimate, by the year 2050 antimicrobial resistant infections might lead to 10 million human deaths (de Kraker et al., 2016).

Antimicrobial resistance (AMR) is a complex phenomenon. Several mechanisms underlying bacterial AMR have been explained, including genetic change(s) or mutations in the target genes, horizontal acquisition of AMR genes, reduced cell wall permeability, efflux pumps etc. (Reygaert, 2018; Martinez and Baquero, 2000). Most of these mechanisms are linked with changes in the bacterial genomes and have been extensively studied using PCR-amplification of the resistance gene(s) and, more recently by whole genome sequencing. However, genomic analyses cannot predict the levels of expressed genes and proteins. Genomes express themselves via the transcriptomes and proteomes, which are the ultimate functional moieties of the cell. But their expression levels fluctuate with external stimuli like environmental stresses and drug exposure (Freiberg et al., 2004; Martínez and Rojo, 2011). Recent reports have shown that alterations in the metabolic pathways is a widely used strategy adopted by bacteria to exhibit drug tolerance, persistence and resistance (Martínez and Rojo, 2011; Bhargava and Collins, 2015; Meylan et al., 2017; Koeva et al., 2017; Goossens et al., 2020). However, the specific metabolism-related protein profiles of drug-resistant bacteria are still poorly understood. During drug exposure, bacteria might adopt alternative cellular functions and/or over- or under express various genes to overcome the drug effect. Thus, the knowledge of transcriptome and proteome of drug-resistant pathogens is necessary for a comprehensive understanding of microbial drug resistance, identification of novel drug targets and for development of new drug molecules. Studies based on elucidating the role of microbial transcriptomes and proteomes in drug resistance are quite less (Singhal et al., 2012; Chopra et al., 2013; Thai et al., 2017; Shen et al., 2021; Pan et al., 2023). Analysis of differentially expressed genes and proteins of drug-resistant bacteria can reveal novel strategies adopted by the bacteria to overcome the effects of drugs.

Thus, the aim of this study was to conduct a combined comparative transcriptomic and proteomic analyses of drug sensitive and -resistant strains of enteropathogenic *E. coli* to understand the mechanisms and biological pathways underlying bacterial multidrug resistance. Two strains of phylogroup D of *E. coli* isolated earlier by us and preserved as glycerol stocks (50% v/v) in a −80°C deep refrigerator were investigated (Bajaj et al., 2015). Molecular typing of these strains in our laboratory had revealed that these strains belonged to the same genomic clade (Bajaj et al., 2015). Also, whole genome analysis of these strains revealed that they were genetically similar as their average nucleotide identity (ANI) was 97.21% (Aswal et al., 2023). *E. coli* IP9 was drug-sensitive while *E. coli* IPE was multidrug resistant exhibiting resistance for many *β*-lactams, fluoroquinolones and kanamycin (Bajaj et al., 2015; Singh et al., 2022). The differentially expressed genes (DEGs) and differentially expressed proteins (DEPs) of multidrug resistant (MDR) *E. coli* were discerned using RNA-sequencing (RNA-Seq) and SWATH-LC MS/MS, respectively. The genes which were observed to be differentially expressed at both mRNA and protein levels were identified and their biological functions and metabolic pathways were elucidated with help of ontological analysis using GO-term enrichment, Cluster of Orthologous Genes (COG)-classification and Kyoto Encyclopaedia of Genes and Genomes (KEGG) pathway analysis. Protein–protein interaction (PPI) networks of the proteins corresponding to these genes were determined to discern their interactome, followed by identification of the hub proteins. To the best of our knowledge, this is the first study which has used an integrated proteomics, transcriptomic and bioinformatics approach to understand the mechanistic details of drug resistance and identify novel drug targets against MDR *E. coli.*

## Materials and methods

### Bacterial strains

In this study, *E. coli* strains - *E. coli* IP9 and *E. coli* IPE isolated earlier from an urban river of India and preserved as glycerol stocks (50% v/v) in a −80°C deep refrigerator were investigated (Bajaj et al., 2015). Triplex-PCR based typing had revealed that these strains belonged to phylogroup D and were of the same genomic clade as revealed by REP-, ERIC- and BOX-PCR based genotypic fingerprinting (Bajaj et al., 2015). *E. coli* IP9 was a drug sensitive isolate while *E. coli* IPE was a MDR strain exhibiting resistance for many *β*-lactams, fluoroquinolones and kanamycin (Bajaj et al., 2015; Singh et al., 2022). The antibiotic susceptibilities of these strains were earlier determined by the Kirby-Bauer disk diffusion susceptibility test and confirmed by PCR-amplification of the relevant genes (Bajaj et al., 2015; Singh et al., 2022). Recently, whole genome sequences of these strains were determined and submitted to the NCBI genome database with accession numbers: GCA_026183935.1 for *E. coli* IPE and GCA_026183955.1 for *E. coli* IP9 (Aswal et al., 2023). The ANI of *E. coli* IP9 and IPE was 97.21%, indicating a significant similarity in their genomes. These strains were revived in our laboratory by overnight incubation in LB broth at 37°C, 200 rpm. The cells were harvested by centrifugation at 8000 rpm for 10 min (at 4°C) from the exponential phase (OD_600_ = 0.8) cultures.

### Transcriptomic analysis

#### RNA isolation, library preparation, and sequencing

Bacteria were collected by centrifugation at 4, 472 x g followed by washing the bacterial pellet with 1X PBS solution. RNA was isolated from both the strains using the Qiagen RNeasy mini kit following the manufacturer’s instructions. Briefly, a cell lysis buffer was added to the cell pellet, mixed by vigorous shaking for 10 min followed by addition of an equal volume of 100% ethanol. The samples were then transferred on a RNeasy micro column for “on-column” DNase treatment. The RNA was then eluted in 20 μL of nuclease-free water. The library preparation was performed using Illumina specific adaptors. The adaptor ligated products were barcoded and 12 cycles of PCR was performed. The PCR products were cleaned using AMPure XP beads. The final enriched library was eluted in 15 μL of 0.1X TE buffer. Sequencing was performed on a Novaseq 6,000 using 150PE chemistry. Paired end sequencing of the transcriptomes of the three biological replicates of the MDR (*E. coli* IPE) and drug sensitive (*E. coli* IP9) strains was done with a read length of 150.

#### Analysis of RNA-Seq data

Nf-core/rnaseq (ver. 3.11.2)^1^ module of the nf-core project was used to analyze the transcriptomics data. Nf-core/rnaseq incorporates several tools for analysis. FastQC (ver. v0.11.9) was used to determine the quality of the sequencing reads. Adapter trimming was done using Trim Galore (ver. 0.6.7). SortMeRNA (ver. 4.3.4) was used to remove ribosomal RNA reads. RSEM (ver. 1.3.1), SALMON (ver. 1.10.1) and HISTAT2 (ver. 2.2.1) were used to map the sequencing reads on the reference genome of *E. coli* K-12 substrain MG1655 (RefSeq assembly; accession GCF_000005845.2). Since this study involved analysis of prokaryotic RNA-Seq data, the parameters used were *--featurecounts_feature_type* = CDS, −-featurecounts_group_type = gene and --skip_rseqc. RNA-Seq data quality control was performed using RSeQC (ver. 3.0.1). RNA-Seq specific quality control (QC) metrics, such as sequencing depth, read distribution and coverage uniformity was performed with RSeQC (ver. 3.0.1) and feature-wise read counting was done using subread feature Counts (ver. 2.0.1). The comprehensive QC report was prepared using MultiQC (ver. 1.13).^2^

Analysis of the DEGs of the MDR and -sensitive strain was performed using the R language and iDEP RNA-Seq differential gene expression pipeline (release 0.96) of the DESeq2 (ver. 1.36.0). Genes were considered differentially expressed when the Benjamini–Hochberg multiple testing adjusted *p*-value was lesser than 0.05 (padj ≤0.05). Genes whose log2 fold change (log2FC) values were > 1.0 or < −1.0 were considered as Differentially Expressed Genes (DEGs).

### Proteomic analysis

#### Protein sample preparation

Bacterial cells were washed with normal saline solution and dispersed in sonication buffer containing 50 mM Tris–HCl,10 mM MgCl_2_, 0.1% sodium azide, 1 mM phenylmethylsulphonyl fluoride and 1 mM; pH 7.4 (Singhal et al., 2012). Cells at a concentration of 1 g wet weight /5 mL of lysis buffer were broken down using a sonicator at 35% amplitude for 10 min at 4°C. The homogenate was centrifuged at 12,000 x*g* for 30 min at 4°C and the clear supernatant was overnight precipitated at -20^ο^ C with chilled acetone. The precipitated protein was collected by centrifugation (12,000 xg, 20 min), air dried and suspended in appropriate volume of protein dissolving buffer. Protein concentration was determined using the Bradford assay (Bradford, 1976).

#### Separation and identification of proteins by nanoLC AB Sciex Triple TOF 5600-MS

Equal amount of protein samples was digested with trypsin and analyzed using a Triple TOF 5600 mass spectrometer (AB Sciex, USA) equipped with Eskigent MicroLC 200 system (Eskigent, Dublin, CA) with an Eskigent C18 - reverse phase column. 1 microgram of digested proteins was desalted online using the online C18 trap column with 98% water, 2% acetonitrile and 0.1% formic acid at flow rate of 5 μL/min for 10 min. The desalted peptides were eluted on a C18 reverse phase analytical column for separation and analysis. A 120 min gradient in multiple steps (ranging from 5–50% acetonitrile in water containing 0.1% formic acid) was set for eluting the peptides from the ChromXP analytical column. The separated peptides were ionized and entered into the mass spectrometer and multiply charged molecules were fragmented using the IDA™ (information dependent data acquisition) criteria of the analyst software for library generation. In brief, 500 ng of all the samples were pooled together and run using IDA criteria of the mass spectrometer for library generation in triplicate. Mass spectrometric data for the first quadrupole was acquired in the range of 350 Da to 1,250 Da whereas 20 most abundant multiply charged peptides were fragmented in the mass range of 150 Da to 1,500 Da in the second quadrupole or collision induced dissociation cell. The accumulation time for each MS/MS experiment was 50 ms. The ionization potential for the turbo V ion source was kept at 4500 V and temperature for source was set 150°C, GS1 and GS2 were at 19 and 15 L/min, respectively. Declustering potential (DP) was set at 80 V. The resultant IDA data files were analyzed in ProteinPilot™ (Sciex software) for identification of peptides and proteins against *E. coli* proteome using Paragon algorithm and the pooled peptide list was used as the spectral library for SWATH analysis. Experiments were performed in technical replicates.

#### SWATH parameters for label free quantification

In SWATH™ Sciex (Sequential window acquisition of all theoretical Spectra) DIA (Data independent acquisition) acquisition method, fixed value Q1 transmission window was kept at 12 Da for the mass range of 350–1,250 Da. Total 75 sequential windows were acquired independently with an accumulation time of 62 ms, along with three technical replicates for each of the sets. Total cycle time was kept constant at <5 s. For label free quantification, peak extraction and spectral alignment was performed using PeakView® 2.2 software with the following parameters: number of peptides selected for quantitation 2, confidence of peptide identification was set as more than 95%, number of fragment ions for each peptide was set as 5, extraction ion chromatogram (XIC) peak width was fixed 30 ppm for matching the RT whereas the XIC extraction window set for matching the peptide across the different samples was set at 5 min. For statistical interpretation, MarkerView software™ (ver. 1.3. 1; AB Sciex) was used. The SWATH acquisition data was processed using SWATH™ Acquisition MicroApp (ver. 2.0) in PeakView® software.

### Data analysis

Raw data was search processed with ProteinPilot™ using the Paragon and Progroup Algorithms for protein and peptide identification. Analysis was also done using the integrated tools in Protein Pilot at 1% false discovery rate (FDR). The identification file was used as spectral library in the quantitation experiment for SWATH/DIA. Retention time calibration was performed using the most abundant peptide across all the samples. Proteins whose log2 fold change (log2FC) values were > 0.5 or < −0.5 (padj ≤0.05) were considered as differentially expressed proteins (DEPs). Both DEGs and DEPs were visualized using ggVolcanoR.^3^

#### GO-term enrichment analysis of DEGs and DEPs

To elucidate the functions of DEGs and DEPs, GO-term enrichment analysis was performed with ShinyGO^4^ using *E. coli* K-12 MG1655 as the reference strain. The enrichment analysis was done at all the three levels of GO-terms: biological process (BP), cellular component (CC) and molecular function (MF). Gene sets that ranked in the top ten for BP, CC and MF were individually enriched for DEGs and DEPs. This data was used to construct GO plots, which show the number of genes and their relative enrichment significance for each enriched category.

#### Identification of the genes differentially expressed at both mRNA and protein levels, GO-term enrichment, COG and KEGG pathway analysis

The concordance in differentially expressed mRNA transcripts and proteins of the MDR strain was evaluated by creating a nine-quadrant correlation plot for DEGs with log2FC expression >0.1 or < −0.1 and for DEPs with log2FC expression >0.5 or < −0.5 (*p* ≤ 0.05). The biological functions and metabolic pathways of the concordantly differentially expressed genes were determined using the GO-term enrichment, COG classification and KEGG pathway analysis. For GO-term enrichment ShinyGO (*p* ≤ 0.05) was used, for COG classification COGclassifier (v1.0.5) was used and for KEGG pathway analysis Database for Annotation, Visualization and Integrated Discovery (DAVID) was used.

#### Protein–protein interactions of the genes differentially expressed at both mRNA and protein levels and identification of the hub proteins

The protein–protein interactions (PPIs) in the concordantly differentially expressed genes were discerned using STRING database (ver.12.0^5^) with ≥0.3 as the threshold strength. The top ten hub proteins were identified using the Maximal Clique Centrality (MCC) algorithm which is reportedly the most effective method of finding hub nodes. The hub proteins were discerned from the PPI network using cytoHubba plugin of Cytoscape ver. 3.9.1.^6^

#### Validation of hub proteins as probable drug targets against MDR *Escherichia coli*

The similarity of the hub proteins with the human proteins was determined using NCBI-BLAST. Hub proteins exhibiting ≥30% sequence identity and ≥ 80% coverage with human proteins, were considered as homologs of the human proteins and were removed from further analysis. The remaining hub proteins were considered as non-homologous to human proteins (NHHPs; Garg et al., 2020).

#### PPI interaction network of antibiotic resistance genes of MDR *Escherichia coli* and the hub proteins

The antibiotic resistance genes of MDR *E. coli* were discerned using the Comprehensive Antibiotic Resistance Database (CARD; McArthur et al., 2013). A PPI network of the proteins encoded by the antibiotic resistance genes and the hub proteins was generated using the STRING database to generate their interactome (at an interaction score of 0.3). The interactome was analyzed using k-mean clustering and the clusters were enriched for biological functions at FDR < =0.05.

## Results

### RNA-Seq data analysis

**Figure 1:**
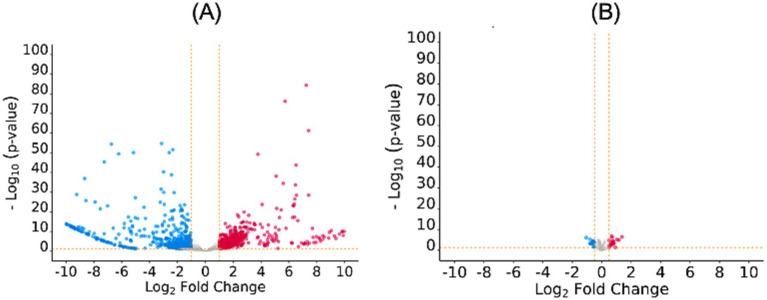
Volcano plots depicting differential gene/protein expressions at log2FC beyond the defined threshold; *p*-value ≤0.05: **(A)** DEGs with log2FC values beyond the range −1 to 1, **(B)** DEPs with log2FC values beyond the range − 0.5 to 0.5. The over- and under-expressed genes/proteins are shown in red and blue color, respectively. The plots were generated using ggvolcanoR (https://ggvolcanor.erc.monash.edu/).

Analysis of RNA-Seq data of the MDR and drug-sensitive *E. coli* strains revealed that 4,101 genes were differentially expressed. Of these, the log2FC value of 2,127 genes was beyond the defined threshold (log2FC > 0.1 for overexpressed genes and log2FC < −0.1 for unexpressed genes; *p* ≤ 0.05). Hence, 2,127 genes were considered as DEGs and analysed further. Of these, 982 genes were found to be overexpressed, while 1,145 genes were found to be under expressed by the *E. coli* MDR strain (Figure 1A; Supplementary Table S1).

### SWATH-LC MS data analysis

Analysis of the SWATH-LC MS data of the MDR and drug-sensitive *E. coli* strains revealed that 770 proteins were differentially expressed. Of these, the log2FC values of 270 proteins were beyond the defined threshold (log2FC > 0.5 for upregulated proteins and log2FC < −0.5 for downregulated proteins; p ≤ 0.05) hence, only these proteins were considered as DEPs and included in further analysis. Of these, 89 proteins were found to be upregulated, while 187 proteins were found to be downregulated by the *E. coli* MDR strain (Figure 1B; Supplementary Table S2).

### GO-term enrichment analysis of the DEGs and DEPs

The overexpressed DEGs were found to be involved in the biological processes related to amide biosynthesis (112 proteins), peptide metabolic process (100 proteins), translation (97 proteins), peptide biosynthesis (96 proteins), ribonucleoprotein complex biogenesis (69 proteins), organelle assembly (53 proteins), ribosome assembly (48 proteins) and ribonucleoprotein complex (43 proteins). The overexpressed DEGs were located within the ribosome (59 proteins), ribonucleoprotein complex (56 proteins), ribosomal subunit (54 proteins), large ribosomal subunit (32 proteins), small ribosomal subunit (22 proteins) and exonuclease repair complex (4 proteins). The molecular functions of the overexpressed DEGs were primarily related to structural molecular activity (59 proteins), structural constituent of ribosome (56 proteins), rRNA binding (52 proteins), tRNA binding (31 proteins), aminoacyl-tRNA ligase activity (20 proteins), ribosome binding (20 proteins), translation regulator activity (18 proteins) (Supplementary Figure S1A). The under expressed DEGs were found to be involved in biological processes related to carboxylic acid catabolic process (100 proteins), carbohydrate transport (79 proteins), monocarboxylic acid catabolic process (58 proteins), response to xenobiotic stimuli (26 proteins) and primary amino acid metabolic processes (18 proteins). They were located within the cell projection (46 proteins), bacterial-type flagella (24 proteins), pilus (22 proteins), bacterial-type flagella basal body (18 proteins), and type II protein secretion system (11 proteins). Their molecular functions were primarily related to carbohydrate transmembrane transporter activity (52 proteins), protein-phosphocysteine-sugar phosphotransferase activity (18 proteins), carbohydrate cation symporter activity (15 proteins), motor activity (8 proteins) and CoA-transferase activity (7 proteins) (Supplementary Figure S1B).

Overexpressed DEPs were found to be involved in biological processes related to amide biosynthesis process (12 proteins). They were located in intracellular regions (56 proteins), cytoplasm (55 proteins) and cytosol (53 proteins). Their molecular functions were related to RNA binding (11 proteins), OB-fold nucleic acid binding domain (3 proteins), polyketide sugar unit biosynthesis (3 proteins) and tRNA synthetase class II (3 proteins) (Supplementary Figure S1C). Under expressed DEPs were involved in biological processes related to organonitrogen compound biosynthesis process (49 proteins), cellular protein metabolic process (33 proteins), small molecule biosynthesis process (31 proteins), cellular amide metabolic process (28 proteins), amide biosynthesis (23 proteins), peptide metabolic process (21 proteins), peptide biosynthesis (19 proteins), translation (19 proteins), fatty acid biosynthesis (7 proteins) and mannose transmembrane transport (3 proteins). They were located within the ribosomal subunit (11 proteins), cytosolic ribosome (11 proteins), large ribosomal unit (6 proteins), small ribosomal subunit (5 proteins), NADH dehydrogenase complex (4 proteins), respiratory chain complex (4 proteins), ATPase complex (3 proteins) and acetyl-CoA carboxylase (2 proteins). Their molecular functions were related to protein binding (51 proteins), ligase activity (18 proteins), rRNA binding (11 proteins), iron ion binding (14 proteins), structural constituent of ribosome (10 proteins), ferrous ion binding (8 proteins) and mannose transmembrane transporter (3 proteins) (Supplementary Figure S1D).

### Discerning the concordance in transcriptomics and proteomics data

**Figure 2:**
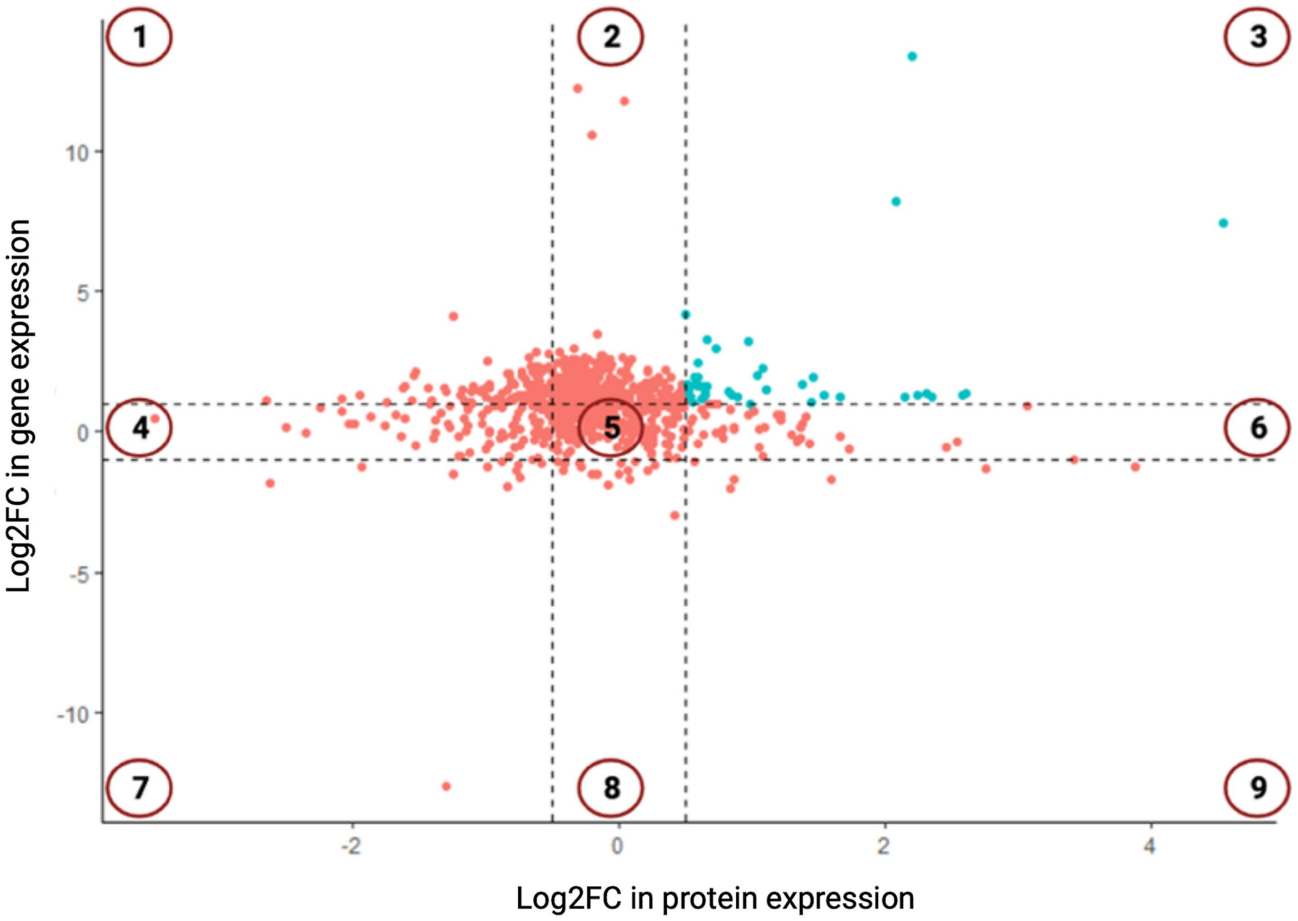
The nine-quadrant plot showing concordance in the log2FC values of differentially expressed genes (DEGs) and differentially expressed proteins (DEPs). Genes which show positive correlation at both mRNA and protein levels are present in 3rd and 7th quadrants and are shown in blue color.

A total of 763 genes/proteins were discerned which exhibited the log2FC differential expression values within the defined thresholds (Supplementary Table S2). The Pearson’s correlation coefficient in the log2FC expression values of transcriptomics and proteomics datasets was 0.078 (*p*-value = 0). Of these 763 genes/proteins, 101 genes/proteins were present in 1st and 9th quadrants suggesting a non-concordance in the expression levels of mRNA and their corresponding proteins. The expression of the genes present in the 1st quadrant was upregulated at mRNA level but downregulated at the protein level. While the expression of the genes present in the 9th quadrant was upregulated at protein level but downregulated at the mRNA level. In the 2nd and the 8th quadrants, 283 genes were present which were significantly differentially expressed at the mRNA level but did not exhibit any differential expression at the protein level. In the 4th and the 6th quadrants, 120 genes were present which did not exhibit any significant differential expression at mRNA level but a significant differential expression at the protein level. In the 3rd and the 7th quadrants, 52 genes were present which exhibited a positive correlation at both transcription and translation levels (Figure 2). Thus, 52 genes were discerned in the *E. coli* MDR strain which were differentially expressed at both mRNA and protein levels. Of these, 41 genes were found to be overexpressed (3rd quadrant, Table 1) and 11 genes were under expressed (7th quadrant, Table 2).

**Table 1:** Details of the concordantly overexpressed genes of *E. coli* MDR strain discerned by combined transcriptomics and proteomics.

S. No	Name of the gene and protein	Function	Log2FC (gene expression)	Log2FC (protein expression)
1.	rfbB (dTDP-glucose 4,6-dehydratase 1 (EC 4.2.1.46))	NAD binding	13.3722738	2.20862609
2.	rfbA (Glucose-1-phosphate thymidylyltransferase 1 (G1P-TT 1) (EC 2.7.7.24) (dTDP-glucose pyrophosphorylase 1) (dTDP-glucose synthase 1))	Metal ion binding	8.17958198	2.0911649
3.	lysU (Lysine--tRNA ligase, heat inducible (EC 6.1.1.6) (Lysyl-tRNA synthetase) (LysRS))	Lysine-tRNA ligase activity	7.4180544	4.55160903
4.	yagU (Inner membrane protein YagU)	Response to acidic pH	4.19006328	0.50106214
5.	rib (3,4-dihydroxy-2-butanone 4-phosphate synthase (DHBP synthase) (DHBPS) (EC 4.1.99.12))	Protein homodimerization activity	3.27334576	0.66636193
6.	sodB (Superoxide dismutase [Fe] (EC 1.15.1.1))	Superoxide dismutase activity	3.21026214	0.97327487
7.	rpsR (Small ribosomal subunit protein bS18 (30S ribosomal protein S18))	Small ribosomal subunit rRNA binding	2.93529538	0.73218065
8.	purU (Formyltetrahydrofolate deformylase (EC 3.5.1.10) (Formyl-FH(4) hydrolase))	Formyltetrahydrofolate deformylase activity	2.44073618	0.60018293
9.	acrA (Multidrug efflux pump subunit AcrA (AcrAB-TolC multidrug efflux pump subunit AcrA) (Acridine resistance protein A))	Transmembrane transporter activity	2.24830789	1.09033838
10.	rpsI (Small ribosomal subunit protein uS9 (30S ribosomal protein S9))	tRNA binding	1.98689822	1.04326103
11.	yhbY (RNA-binding protein YhbY)	RNA binding	1.95772549	1.46607357
12.	eno (Enolase (EC 4.2.1.11) (2-phospho-D-glycerate hydro-lyase) (2-phosphoglycerate dehydratase))	Protein homodimerization activity	1.94705179	0.57238355
13.	glnH (Glutamine-binding periplasmic protein (GlnBP))	Glutamine binding	1.91812861	0.60066724
14.	greA (Transcription elongation factor GreA (Transcript cleavage factor GreA))	Bacterial-typeRNA polymerase holoenzyme binding	1.70603887	0.58989593
15.	rpmG (Large ribosomal subunit protein bL33 (50S ribosomal protein L33))	Structural constituent of ribosome	1.70091029	0.52116122
16.	dacA (D-alanyl-D-alanine carboxypeptidase DacA (DD-carboxypeptidase) (DD-peptidase) (EC 3.4.16.4) (Beta-lactamase) (EC 3.5.2.6) (Penicillin-binding protein 5) (PBP-5))	Beta-lactamase activity	1.69712814	0.5918107
17.	nuoB (NADH-quinone oxidoreductase subunit B (EC 7.1.1.-) (NADH dehydrogenase I subunit B) (NDH-1 subunit B) (NUO2))	Quinone binding	1.68110768	1.38351941
18.	accC (Biotin carboxylase (EC 6.3.4.14) (Acetyl-coenzyme A carboxylase biotin carboxylase subunit A))	Acetyl-CoA carboxylase activity	1.63999445	0.62329811
19.	topA (DNA topoisomerase 1 (EC 5.6.2.1) (DNA topoisomerase I) (Omega-protein) (Relaxing enzyme) (Swivelase) (Untwisting enzyme))	DNA binding	1.63075652	0.6043047
20.	tolB (Tol-Pal system protein TolB)	Protein-containing complex binding	1.61881347	0.66940982
21.	sdhB (Succinate dehydrogenase iron–sulfur subunit (EC 1.3.5.1))	Succinate dehydrogenase (ubiquinone) activity	1.57664473	0.52821973
22.	grxD (Glutaredoxin 4 (Grx4) (Monothiol glutaredoxin))	Disulfide oxidoreductase activity	1.47892511	1.10904147
23.	thiI (tRNA sulfurtransferase (EC 2.8.1.4) (Sulfur carrier protein ThiS sulfurtransferase) (Thiamine biosynthesis protein ThiI) (tRNA 4-thiouridine synthase))	tRNA U4 sulfurtransferase	1.4562179	0.83577373
24.	rlmB (23S rRNA (guanosine-2’-O-)-methyltransferase RlmB (EC 2.1.1.185) (23S rRNA (guanosine2251 2’-O)-methyltransferase) (23S rRNA Gm2251 2’-O-methyltransferase))	RNA binding	1.43916572	0.65337225
25.	rluD (Ribosomal large subunit pseudouridine synthase D (EC 5.4.99.23) (23S rRNA pseudouridine(1911/1915/1917) synthase) (rRNA pseudouridylate synthase D) (rRNA-uridine isomerase D))	Pseudouridine synthase activity	1.38208643	0.52671278
26.	bamA (Outer membrane protein assembly factor BamA (Omp85))	Outer membrane biogenesis	1.3670567	2.61384269
27.	smpB (SsrA-binding protein (Small protein B))	RNA binding	1.34409894	2.3132047
28.	seqA (Negative modulator of initiation of replication)	DNA replication origin binding	1.31627878	2.24904635
29.	gstA (Glutathione S-transferase GstA (EC 2.5.1.18) (GST B1-1))	Glutathione transferase activity	1.31476427	1.53911607
30.	nagA (N-acetylglucosamine-6-phosphate deacetylase (GlcNAc 6-P deacetylase) (EC 3.5.1.25))	N-acetylglucosamine-6-phosphate deacetylase activity	1.30408659	0.65904607
31.	valS (Valine--tRNA ligase (EC 6.1.1.9) (Valyl-tRNA synthetase) (ValRS))	Valine-tRNA ligase activity	1.2939003	2.5908781
32.	degP (Periplasmic serine endoprotease DegP (EC 3.4.21.107) (Heat shock protein DegP) (Protease Do))	Serine-type peptidase activity	1.28686848	0.85463352
33.	aspS (Aspartate--tRNA ligase (EC 6.1.1.12) (Aspartyl-tRNA synthetase) (AspRS))	Aspartate-tRNA ligase activity	1.2654914	1.66674373
34.	ydcH (Uncharacterized protein YdcH)	Transceription regulator	1.26094475	0.89665598
35.	yhbJ (RNase adapter protein RapZ)	RNA binding	1.24343218	2.15306445
36.	rcsF Outer membrane lipoprotein RcsF	Cellular response to cell envelop stress	1.22552494	0.55077488
37.	ppiB (Peptidyl-prolyl cis-trans isomerase B (PPIase B) (EC 5.2.1.8) (Rotamase B))	Peptidyl-prolyl cis-trans isomerase activity	1.2217117	2.3605269
38.	lysS (Lysine--tRNA ligase (EC 6.1.1.6) (Lysyl-tRNA synthetase) (LysRS))	Lysine-tRNA ligase activity	1.20254035	0.62226595
39.	hemL (Glutamate-1-semialdehyde 2,1-aminomutase (GSA) (EC 5.4.3.8) (Glutamate-1-semialdehyde aminotransferase) (GSA-AT))	Transaminase activity	1.07279768	1.45671431
40.	rlmI (Ribosomal RNA large subunit methyltransferase I (EC 2.1.1.191) (23S rRNA m5C1962 methyltransferase) (rRNA (cytosine-C(5)-)-methyltransferase RlmI))	Protein homodimerization activity	1.06900742	0.5624809
41.	yeeZ (Protein YeeZ)	ATP binding	1.00468423	0.99181048

**Table 2:** Details of the concordantly under-expressed genes of *E. coli* MDR strain discerned by combined transcriptomics and proteomics.

S. No	Name of the gene and protein	Function	Log2FC (gene expression)	Log2FC (protein expression)
1	metE (5-methyltetrahydropteroyltriglutamate--homocysteine methyltransferase (EC 2.1.1.14) (Cobalamin-independent methionine synthase) (Methionine synthase, vitamin-B12 independent isozyme))	Methionine synthase activity	−2.6135055	−1.8460639
2	yrdA (Protein YrdA)	Zinc ion binding	−1.9251177	−1.2546857
3	Bfr (Bacterioferritin (BFR) (EC 1.16.3.1) (Cytochrome b-1) (Cytochrome b-557))	Ferric iron binding	−1.2901495	−12.63613
4	malE (Maltose/maltodextrin-binding periplasmic protein (MMBP) (Maltodextrin-binding protein) (Maltose-binding protein) (MBP))	Maltose binding	−1.2432956	−1.5255921
5	yjiY (Pyruvate/proton symporter BtsT (Brenztraubensaure transporter) (Pyruvate/H(+) symporter))	Symporter activity	−0.9818285	−1.2231589
6	yahK (Aldehyde reductase YahK (EC 1.1.1.2) (Zinc-dependent alcohol dehydrogenase YahK))	Alcohol dehydrogenase (NADP+) activity	−0.8786242	−1.0459354
7	yhbW (Luciferase-like monooxygenase)	Oxidoreductase activity	−0.8385109	−1.9618579
8	cyaY (Iron–sulfur cluster assembly protein CyaY (Bacterial frataxin ortholog))	Ferric iron binding	−0.7704802	−1.409128
9	malM (Maltose operon periplasmic protein)	Carbohydrate transport	−0.7482777	−1.1703698
10	malK (Maltose/maltodextrin import ATP-binding protein MalK (EC 7.5.2.1))	ABC-type maltose transporter activity	−0.7322275	−1.6484885
11	glpQ (Glycerophosphodiester phosphodiesterase, periplasmic (Glycerophosphoryl diester phosphodiesterase, periplasmic) (EC 3.1.4.46))	Calcium ion binding	−0.5756688	−1.1040456

### Functional analysis of the genes exhibiting concordance in differential expression at both mRNA and protein levels

#### GO-term enrichment analysis

**Figure 3:**
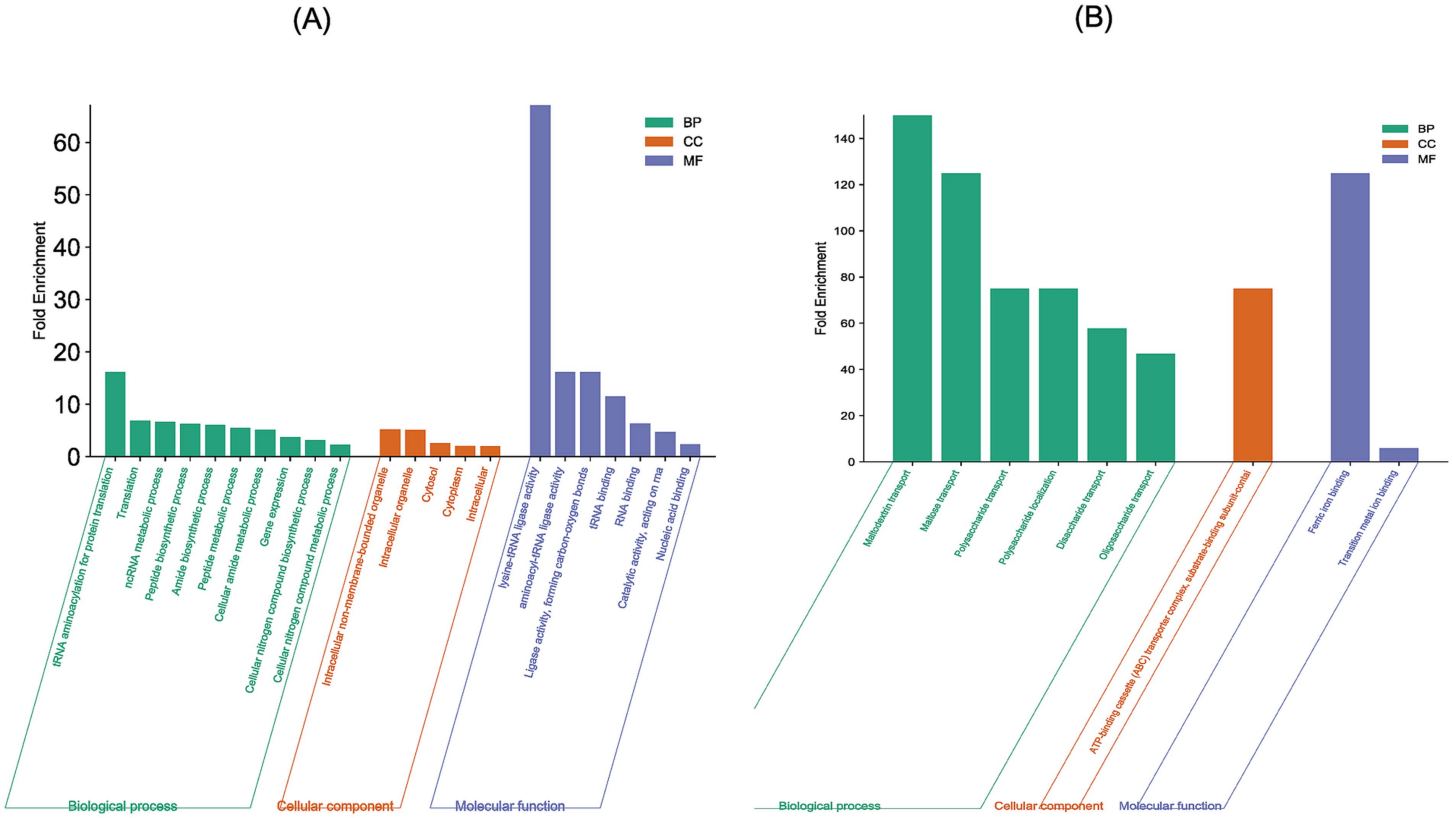
GO-enriched terms associated with the genes exhibiting differential expression at both mRNA and protein levels in *E. coli* MDR strain: **(A)** GO-terms associated with the 41 concordant overexpressed genes **(B)** GO-terms associated with the 10 concordant under-expressed genes. The X-axis shows the GO-terms and the Y-axis shows the fold enrichment values associated with each GO-term.

GO-term enrichment analysis of the 41 genes which exhibited overexpression at both mRNA and protein levels revealed that these were primarily involved in biological processes related to cellular amide metabolic biosynthesis (11 proteins), amide biosynthesis (10 proteins), ncRNA metabolic process (9 proteins), translation (8 proteins), peptide biosynthesis (8 proteins) and tRNA aminoacylation for protein translation (4 proteins). At the molecular functional level, they exhibited nucleic acid binding (16 proteins), RNA binding (12 proteins), tRNA binding (5 proteins), aminoacyl-tRNA ligase activity (4 proteins) and lysine-tRNA ligase activity (2 proteins). They were located in cytoplasm (28 proteins), non-membranous bound organelle (5 proteins) and intracellular organelle (5 proteins) (Figure 3A).

GO-term enrichment analysis of the 11 genes which exhibited under expression at both mRNA and protein levels revealed that these were primarily involved in biological processes related to maltodextrin transport (2 proteins), polysaccharides transport (2 proteins) and oligosaccharide transport (2 proteins). At the molecular functional level, they exhibited transition metal binding activities (5 proteins) and ferric ion binding (2 proteins). All the proteins were located on the ATP-binding cassette transporter complex (Figure 3B).

#### COG classification and KEGG pathway analysis

**Figure 4:**
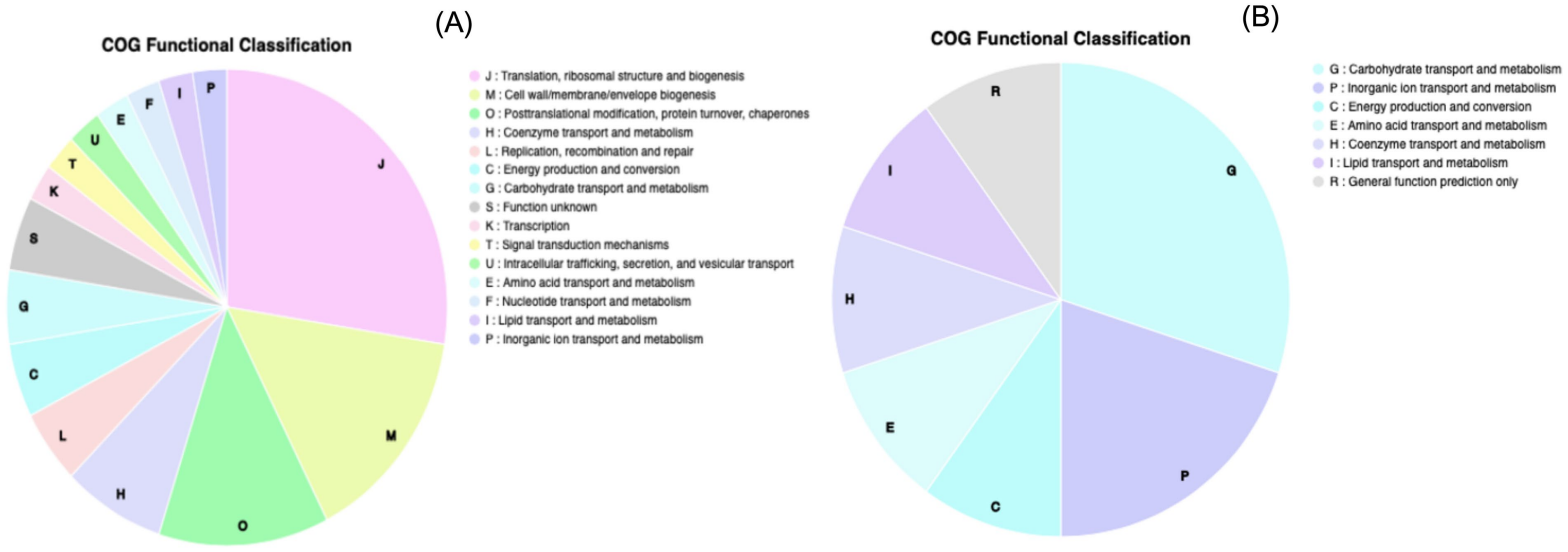
COG-based functional classification of the genes exhibiting differential expression at both mRNA and protein levels in *E. coli* MDR strain: **(A)** overexpressed genes **(B)** under-expressed genes.

COG classification of the 41 genes which exhibited overexpression at both mRNA and protein levels revealed that they belonged to 15 functional categories. Of these, 11 genes were involved in translation, ribosomal structure and biogenesis, six genes in cell wall/membrane/envelope biogenesis, five genes in post-translational modification, turnover and chaperon, three genes in coenzyme transport and metabolism, two genes each in replication, recombination and repair, energy production and conversion, carbohydrate transport and metabolism and unknown function. One gene each was involved in transcription, signal transduction mechanism, intracellular trafficking, secretion and vesicular transport, amino acid transport and metabolism, nucleoside transport and metabolism, lipid transport and metabolism, and inorganic ion transport and metabolism (Figure 4A). The 11 genes which exhibited similarity in under expression at both mRNA and protein levels were classified into seven COG functional categories. Three genes were involved in carbohydrate transport and metabolism, two in inorganic ion transport and metabolism, one each in energy production and conversion, in amino acid transport and metabolism, coenzyme transport and metabolism, lipid transport and metabolism and general function (Figure 4B).

**Figure 5:**
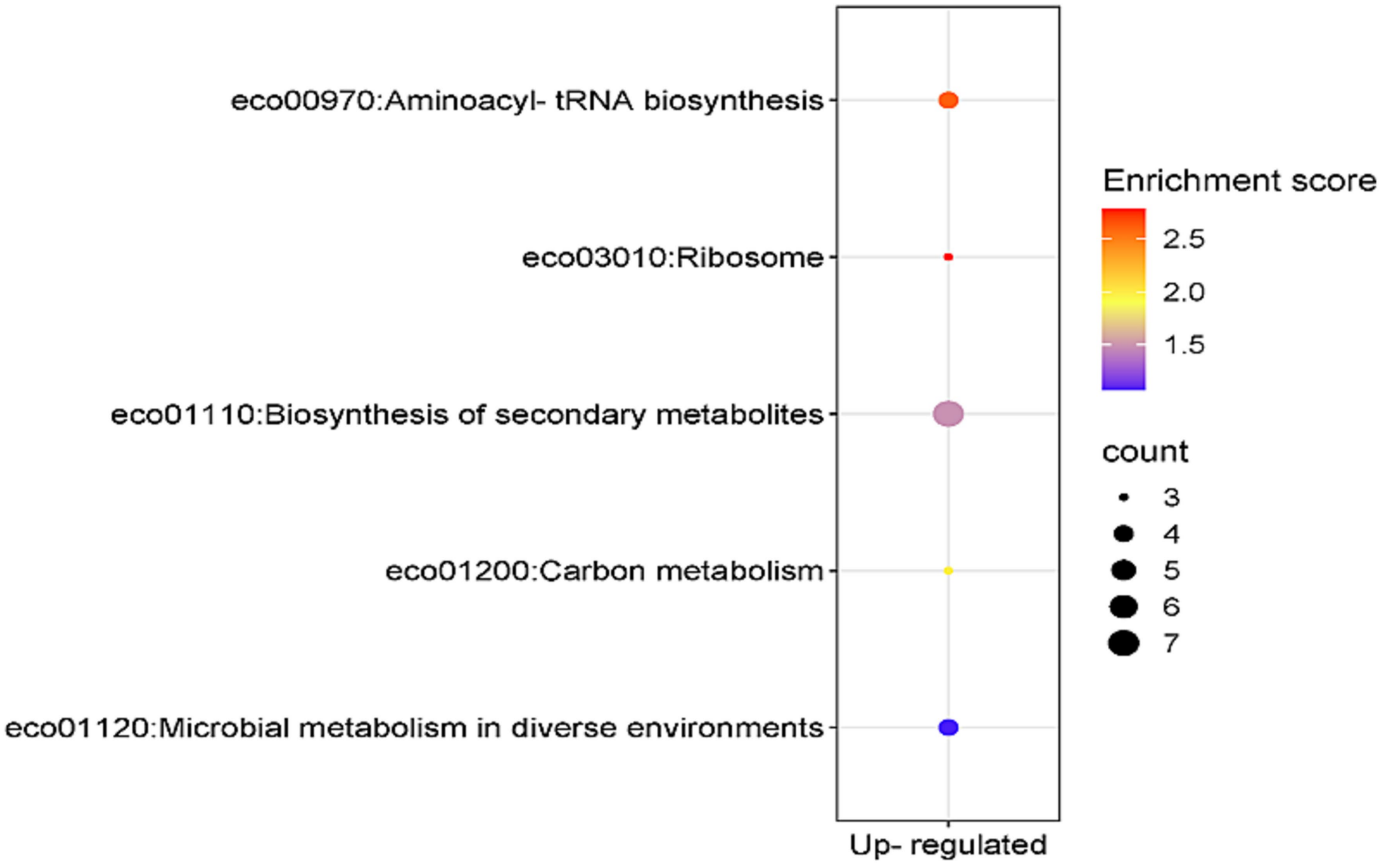
KEGG-enriched pathways and enrichment score associated with the concordantly overexpressed genes in *E. coli* MDR strain.

The KEGG pathway enrichment analysis of the 41 genes which were overexpressed at both mRNA and protein levels revealed that seven of these genes were involved in biosynthesis of secondary metabolites (enrichment score 1.485704), four genes in aminoacyl-tRNA biosynthesis pathway (enrichment score 2.608108) and three genes in ribosome biosynthesis (enrichment score 2.783654) (Figure 5). The KEGG pathway enrichment analysis of the 11 genes concordantly under expressed at both mRNA and protein levels did not yield any result.

#### PPI network analysis and identification of the hub proteins

**Figure 6:**
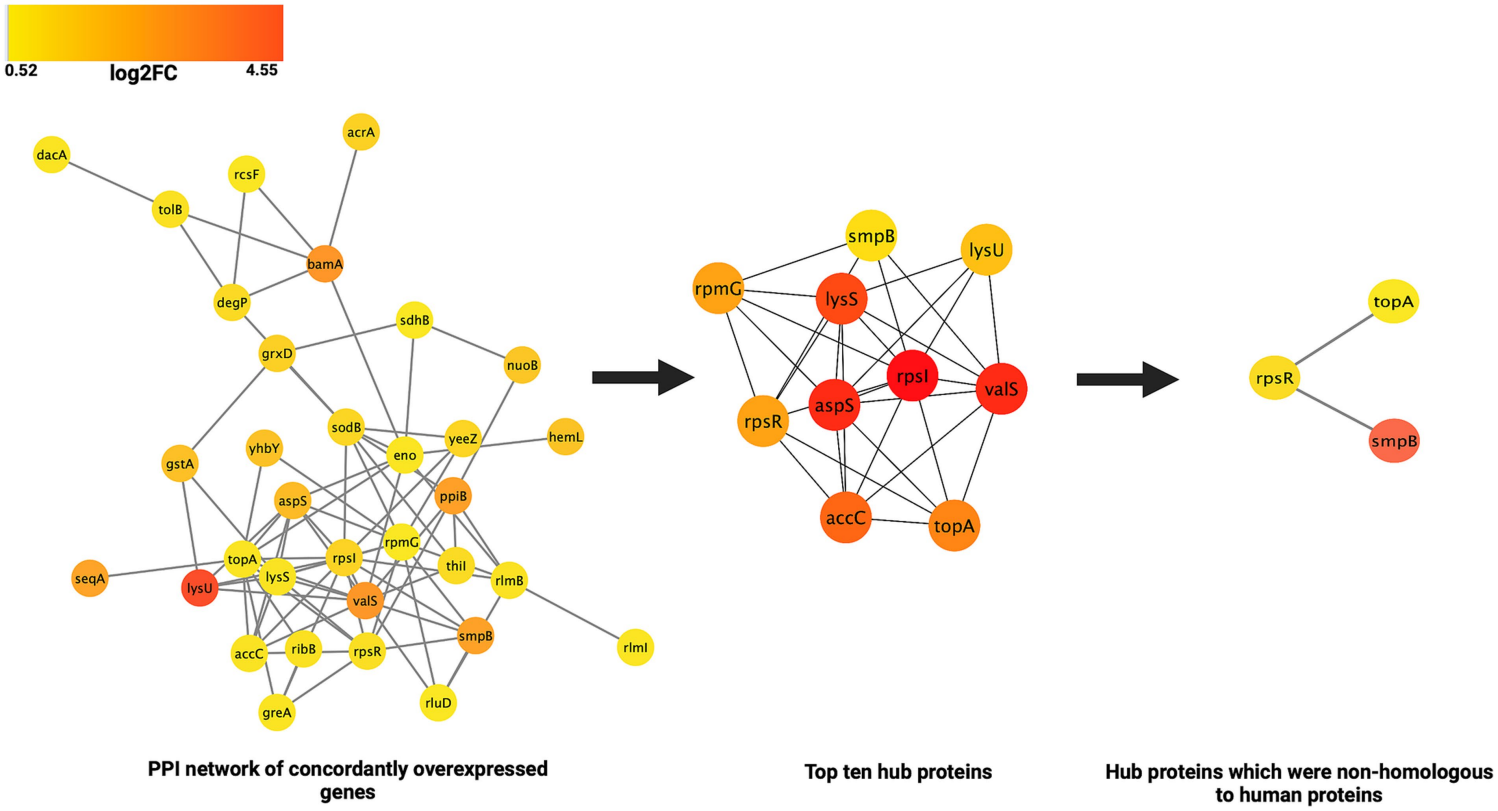
Schematic representation of the PPI networks of the concordantly overexpressed genes, identification of the hub proteins and potential drug targets against MDR *E. coli.*

Analysis of the PPI network of the proteins corresponding to the 41 genes concordantly overexpressed at both transcription and translation levels revealed that they were involved in 81 PPIs (Figure 6). On the basis of the maximal clique centrality (MCC) score, ten proteins were identified as the hub proteins of these interaction networks. These were: aspS (aspartate-tRNA ligase/ aspartyl-tRNA synthetase), valS (valine-tRNA ligase/ valyl-tRNA synthetase), lysS (lysine-tRNA ligase/lysyl-tRNA synthetase), lysU (lysine-tRNA ligase/lysyl-tRNA synthetase), rpmG (large ribosomal subunit protein bL33 (50S ribosomal protein L33)), rpsI (small ribosomal subunit protein uS9/30S ribosomal protein S9), rpsR (small ribosomal subunit protein/30S ribosomal protein S18), spmB (small protein B/SsrA-binding protein), topA (DNA topoisomerase 1) and accC (biotin carboxylase). The hub proteins and their MCC scores have been detailed in Table 3. The hub proteins were found to be primarily involved in tRNA and lysyl-tRNA aminoacylation, and translation. At the molecular level they were involved in tRNA and RNA binding and were located in ribosomal subunits and cytosol. However, no PPI networks were discerned for the proteins corresponding to the concordant under expressed genes.

**Table 3:** Details of the top ten hub proteins discerned from the PPI networks of concordantly overexpressed genes of MDR *E. coli* strain.

Hub protein	Uniprot ID	% identity with human proteins	% alignment with human proteins	Homologous with the human proteins	Log2FC (based on SWATH -LC MS/MS)	Potential drug target
RpsI	P0A7X3	39.02	93	Yes	1.04	No
AspS	P21889	40.19	99	Yes	1.66	No
ValS	P07118	40.08	98	Yes	2.59	No
LysS	P0A8N3	43.35	97	Yes	0.62	No
AccC	P24182	47.69	99	Yes	0.62	No
TopA	P06612	25.28	65	No	0.60	Yes
RpmG	P0A7N9	44.44	81	Yes	0.52	No
RpsR	P0A7T7	-	-	No	0.73	Yes
LysU	P0A8N5	44.07	97	Yes	4.55	No
SmpB	P0A832	-	-	No	2.31	Yes

#### Identification of potential drug target(s) from the hub proteins

To avoid cross-reactivity of the potential inhibitors of the hub proteins with the human proteins, human homologs of the hub proteins were discerned in the human proteome using NCBI-BLAST. Of the ten potential hub proteins which could be potential drug targets, seven hub proteins (rpsI, aspS, valS, lysS, accC, rpmG and lysU) showed homology with the human proteins with identity ranging between 39 to 47% and alignment coverage ranging from 81 to 99%. Two hub proteins rpsR and smpB did not show any homology with the human proteins, while topA showed 25.28% identity and 65% alignment coverage with the human proteins (Table 3).

#### Interactome of the antibiotic resistance genes of MDR *Escherichia coli* with the hub proteins

CARD analysis revealed 69 genes whose products were related to antibiotic resistance in MDR *E. coli* (Supplementary Table S3). Of these, 47 genes could be mapped by STRING on the PPI network along with the hub proteins - topA, smpB and rpsR. Three prominent clusters were observed in the interactome: cluster 1 composed of 39 proteins involved in antibiotic detoxification (acrA, acrB, acrD, acrE, acrF, acrR, acrS, baeR, cpxA, crp, emrA, emrB, emrK, emrY, evgA, evgS, gadX, hha, kdpE, leuO, marA, marR, mdfA, mdtA, mdtB, mdtC, mdtE, mdtF, mdtG, mdtH, mdtM, mdtN, mdtO, mdtP, mprA, soxR, soxS, tolC and ybiH). Cluster2 contained 7 proteins involved in lipid A biosynthesis (eptA, ugd, arnT, arnC, bacA, yojI and msbA) and cluster 3 contained 4 proteins involved in RNA binding (rpsR, smpB, topA and rsmA). The complete interactome has been depicted in Supplementary Figure S2.

## Discussion

An in-depth understanding of AMR is paramount for development of new antibiotics/drugs, vaccines and diagnostic tools. AMR has been mainly studied using standard microbiological growth-inhibition methods, PCR-amplification of the AMR genes, gene sequencing for mutation detection and more recently by whole genome sequencing (Boolchandani et al., 2019). Recently, several studies have indicated that bacteria withstand antibiotic stress by modifying their metabolic pathways (Richter et al., 2017; Stokes et al., 2019). However, the role of microbial metabolic pathways and other proteins in the context of bacterial drug resistance is inadequately understood. In the present study, we have used transcriptomics, proteomics and bioinformatics approaches to identify differentially expressed genes and proteins of MDR *E. coli* followed by extensive bioinformatics-based studies to discern novel drug targets against antibiotic resistant *E. coli*.

The differentially expressed genes of the MDR *E. coli* were inferred by integrating RNA-Seq and SWATH-LC MS/MS datasets to identify genes exhibiting concordant differential expression at both transcriptomics and proteomics levels. Thus, 763 genes/proteins were discerned whose log2FC differential expression values attained the defined thresholds. The results from both the technologies were integrated for unambiguous and technical artifact-free interpretations. The Pearson’s correlation coefficient between the log2FC expression values of transcriptomics and proteomics datasets was 0.078 (*p*-value = 0), which is low. This is, however, not very surprising because several studies have reported that mRNA expression levels and protein level abundance do not necessarily correlate, due to biological differences between transcription and translation processes and, also due to experimental limitations (Gygi et al., 1999; Brötz-Oesterhelt et al., 2005). Moreover, the correlation between mRNA and protein abundance was reportedly not very strong in bacteria (Gygi et al., 2000). From the biology viewpoint, differential RNA and protein turnover, post-translational modifications, proteolytic processing events and allosteric protein interactions might result in a low concordance in mRNA and protein levels. From the technological viewpoint, challenges in the experimental design and data interpretation methods might be responsible for non-concordance in mRNA and protein levels.

On the basis of the nine-quadrant correlation plot, it was discerned that 52 genes of the MDR *E. coli* showed a positive correlation in differential gene expression at both transcription and translation levels. Of these, 41 genes were overexpressed and 11 genes were under expressed by MDR *E. coli* at both mRNA and protein levels. GO-term enrichment analysis of these 41 overexpressed genes revealed that they were primarily involved in biological processes related to amide/peptide/protein biosynthesis and translation. KEGG pathway enrichment analysis of the 41 overexpressed genes revealed that they were primarily involved in biosynthesis of aminoacyl-tRNA, ribosomes and secondary metabolites. Thus, our results are similar to a recent study which reported that proteins predominantly related to metabolism, transcriptional and translational regulation, and stress response showed differential expression in drug resistant *E. coli* and other bacteria (Mehrotra et al., 2023). Thus, inhibitors of amide/peptide/protein biosynthesis and translation can be suitable candidates against drug resistant *E. coli.* However, such drug molecules which inhibit bacterial translation and related processes are available (e.g., aminoglycosides, tetracyclines macrolides etc.) but increasing instances of bacterial resistance against these antibiotics are being reported. Thus, alternative drug targets/strategies need to be discovered.

Investigating the PPIs/interactome not only helps in understanding the function(s) of individual protein(s), it also helps in characterizing the various pathways in which proteins might be involved. Analysis of the PPI networks of the proteins corresponding to the 41 concordantly overexpressed genes of the *E. coli* MDR strain revealed that they were involved in 81 PPI networks. Ten proteins were identified as the hub proteins of these PPI networks. These were: aspS (aspartate-tRNA ligase/ aspartyl-tRNA synthetase), valS (valine-tRNA ligase/ valyl-tRNA synthetase), lysS (lysine-tRNA ligase/lysyl-tRNA synthetase), lysU (lysine-tRNA ligase/lysyl-tRNA synthetase), rpmG (large ribosomal subunit protein bL33 (50S ribosomal protein L33)), rpsI (small ribosomal subunit protein uS9/30S ribosomal protein S9), rpsR (small ribosomal subunit protein/30S ribosomal protein S18), spmB (small protein B/SsrA-binding protein), topA (DNA topoisomerase 1) and accC (biotin carboxylase). At the functional level, the majority of the hub proteins were involved in aminoacylation of tRNA and protein-translation.

It is well known that hub proteins can interact with multiple proteins and they are critically important for the whole biological system due to their high connectivity (Higurashi et al., 2008; Ota et al., 2016). Inhibition/elimination of the function of a hub protein can influence multiple downstream pathways and biological networks which also affects the organismal fitness (He and Zhang, 2006). Thus, blocking the activity of hub proteins with appropriate inhibitors/drugs can be a useful strategy against a pathogen (Garg et al., 2020). This implies that the hub proteins discerned in this study might be explored as potential drug targets against MDR *E. coli*. However, if a homolog of a potential drug target is present in the host, it might lead to cross-reactivity of the inhibitor/drug with the host protein and, consequently undesirable side effects. Therefore, to avoid cross-reactivity of the potential inhibitors of the hub proteins with the host proteins, homology between the human and the hub proteins was discerned by NCBI-BLAST. Of the ten hub proteins, seven hub proteins (rpsI, aspS, valS, lysS, accC, rpmG and lysU) exhibited homology with the human proteins, their identity ranging between 39 to 47% and alignment coverage ranging between 81 to 99%. However, two hub proteins - smpB and rpsR and did not show any homology with the human proteins, while topA showed 25.28% identity and 65% alignment coverage with the human proteins. Hence, the hub proteins smpB, rpsR and topA might be explored as potential drug targets against MDR *E. coli.*

SmpB is reportedly a small protein (B) which along with ssrA (small stable RNA A) is required during the process of bacterial trans-translation for recycling of ribosomes stalled on defective mRNA (Li et al., 2013). In *Neisseria gonorrhoeae*, *Mycobacterium tuberculosis,* and *Staphylococcus aureus* trans-translation was reportedly essential for viability and virulence of bacteria (Personne and Parish, 2014; Liu et al., 2018; Aron et al., 2021). Also, disruption of the trans-translation pathway was reported to result in increased sensitivity of bacteria for antibiotics (Huang et al., 2019; Campos-Silva et al., 2021). Thus, SmpB has been proposed as a potential drug target against several bacteria (Alumasa and Keiler, 2015).

RpsR is a small ribosomal subunit (30S) protein which is an essential component of the protein biosynthetic machinery of *E. coli* (Shoji et al., 2011). Previous studies have reported that the expression of the gene encoding rpsR was greatly enhanced during biofilm formation in *E. coli* (Schembri et al., 2003; Beloin et al., 2004). A PPI network based study based on integration of differentially expressed genes of *E. coli* during stress conditions implicated that many RNA binding proteins (including rpsR) played an important role in bacterial response to stress (Nagar et al., 2016). In *Deinococcus radiodurans,* a highly upregulated expression of the ribosomal proteins rplB, rpsL, rpsR was reported in response to oxidative stress (Gao et al., 2020).

TopA is a DNA topoisomerase I enzyme which regulates global and local DNA supercoiling. DNA supercoiling affects many DNA centred processes such as, DNA replication, recombination, transcription and transposition (Drlica, 1992; Usongo and Drolet, 2014). Interestingly, the levels of DNA supercoiling are dependent on processes like transcription (Liu and Wang, 1987), nutrient availability and growth environment (Goldstein and Drlica, 1984; Balke and Gralla, 1987; Cheung et al., 2003). TopA activity has been reportedly essential for bacterial response to acid exposure, heat shock and antibiotics (Qi et al., 1996; Weinstein-Fischer et al., 2000; Stewart et al., 2005; Yang et al., 2015). Also, topA activity has been shown to be important for adaptation of *E. coli* for high temperature and oxidative stress (Qi et al., 1999; Tse-Dinh, 2000; Weinstein-Fischer et al., 2000).

To summarize, integrative proteo-transcriptomics revealed several genes which were differentially expressed by MDR *E. coli*. Bioinformatic analysis using GO-terms, COG and KEGG annotations revealed that the 41 overexpressed genes were primarily involved in tRNA aminoacylation and peptide and amide biosynthesis. Analysis of the PPI networks of the upregulated proteins revealed ten hub proteins of which, three hub proteins *viz.* smpB, rpsR and topA were non-homologous to human proteins. Several published reports have indicated an important role of these proteins in resistance for antibiotics and stress adaptation in *E. coli*. Moreover, inhibitors of these hub proteins would exhibit novel mechanisms of action hence, emergence of resistance against these seems unlikely in the near future. Further PPI network studies of the antibiotic resistance genes of MDR *E. coli* with the hub proteins also confirmed that smpB, rpsR and topA interacted with several antibiotic resistance genes. Since these hub proteins regulate several biological pathways of *E. coli*, directly and/or indirectly related to antibiotic stress, inhibitors of these proteins can be effective against both drug-resistant and -sensitive populations of *E. coli.* Moreover, non-homology of these proteins with the host proteins, leaves little scope for cross-reactivity with human proteins and undesirable side effects. However, further experiments including a greater number of drug-resistant and -sensitive strains of *E. coli* are required to understand the complex phenomenon as microbial drug resistance and explore the utility of the hub proteins smpB, rpsR and topA as novel drug targets against *E. coli*. Also, a judicious use of inhibitors of these novel drug-targets is warranted to minimize the possibility of resistance emergence against them, in future.

## Data Availability

The transcriptomics datasets of this study are accessible as BioProject PRJNA1066082. *E. coli* IPE (MDR) and *E. coli* IP9 (drug sensitive) raw reads are available with SRA accessions SRR27601718 and SRR27601719, respectively. Proteomics datasets of this study are available via ProteomeXchange with identifier PXD048998. R script to generate 9 quadrant plot to integrate transcriptomics and proteomics data is available in Figshare (Kumar et al., 2024).

## Footnotes

^1^https://github.com/nf-core/rnaseq, accessed on 13 May 2023.

^2^ http://multiqc.info/

^3^ https://ggvolcanor.erc.monash.edu/

^4^ http://bioinformatics.sdstate.edu/go/

^5^ https://cn.string-db.org/

^6^ https://cytoscape.org
