# Supplementary material for: An integrated proteo-transcriptomics approach reveals novel drug targets against multidrug resistant Escherichia coli
Source: Front Microbiol. 2025 Feb 25;16:1531739. doi: 10.3389/fmicb.2025.1531739 (PMC11893563; doi:10.3389/fmicb.2025.1531739)
Supplement: Supplementary file 3 [file Table_3.docx]

**Supplementary Table 3:** **Antibiotic resistance genes discerned using the Comprehensive**

**Antibiotic Resistance Database in MDR *E. coli***

| S.No. | Antibiotic resistance mechanism | Antibiotic resistance genes |
| --- | --- | --- |
| 1. | antibiotic efflux | *gadX, mdtF, mdtE, CRP, AcrF, AcrE, AcrS, TolC, rsmA, emrB, emrA, emrR, acrD, evgS, evgA, emrK, emrY, YojI, mdtC, mdtB, mdtA, KpnE, KpnF, kdpE, mdfA, msbA, mdtG, mdtH, H-NS, marA, mdtN, mdtO, mdtP, cpxA, acrB, acrA, leuO, mdtM, tet(B), qacEdelta1, baeR, AcrAB-TolC, MarR, soxR, soxS, AcrR and tetR* |
| 2. | antibiotic target alteration | *bacA, ArnT, PmrF, ugd, eptA, vanG, AcrAB-TolC, MarR, soxR, soxS, AcrR and tetR* |
| 3. | antibiotic inactivation | *EC-19, CMY-42, catA1, aadA5, Mrx, mphA, APH(6)-Id, APH(3'')-Ib, catB3, OXA-1, AAC(6')-Ib10, TEM-1* |
| 4 | antibiotic target replacement | *sul1, dfrA17, sul2* |
