# Supplementary figures and images for: An integrated proteo-transcriptomics approach reveals novel drug targets against multidrug resistant Escherichia coli
Source: Front Microbiol. 2025 Feb 25;16:1531739. doi: 10.3389/fmicb.2025.1531739 (PMC11893563; doi:10.3389/fmicb.2025.1531739)

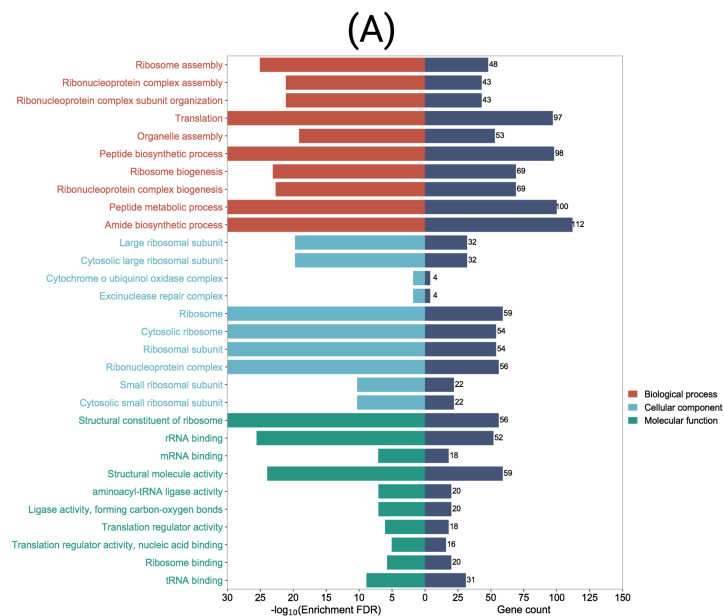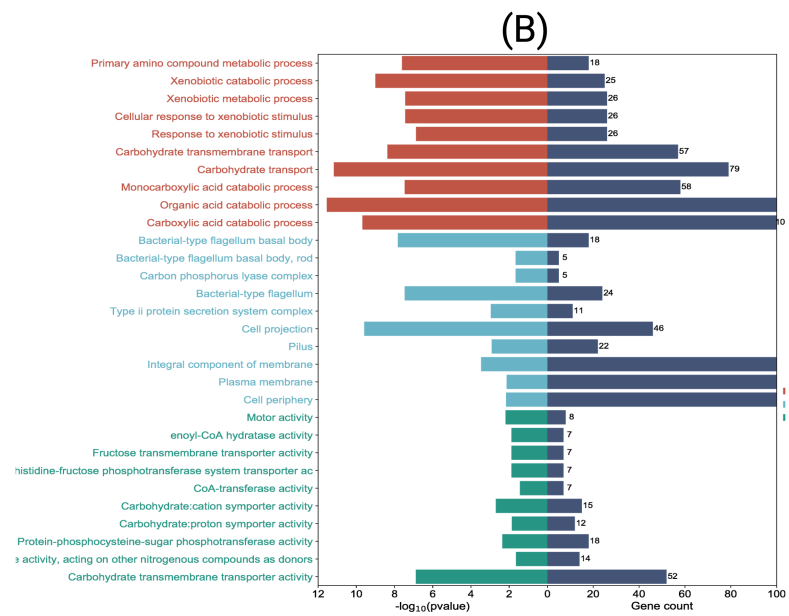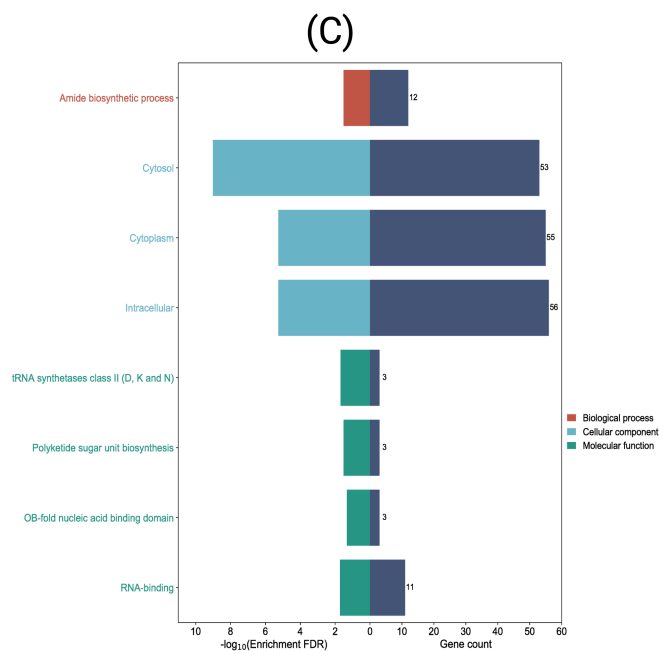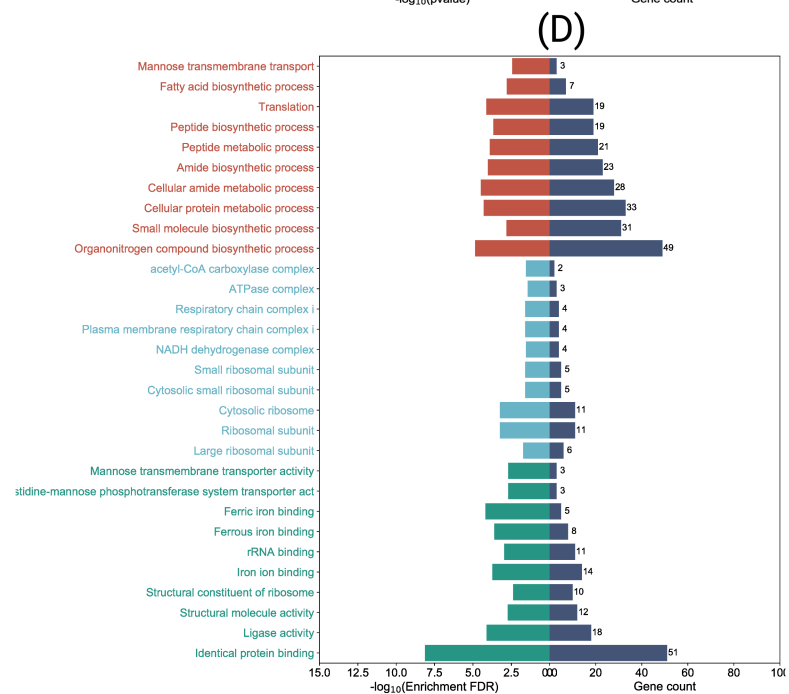

Supplement: Supplementary file 4 [file Data_Sheet_1.pdf]

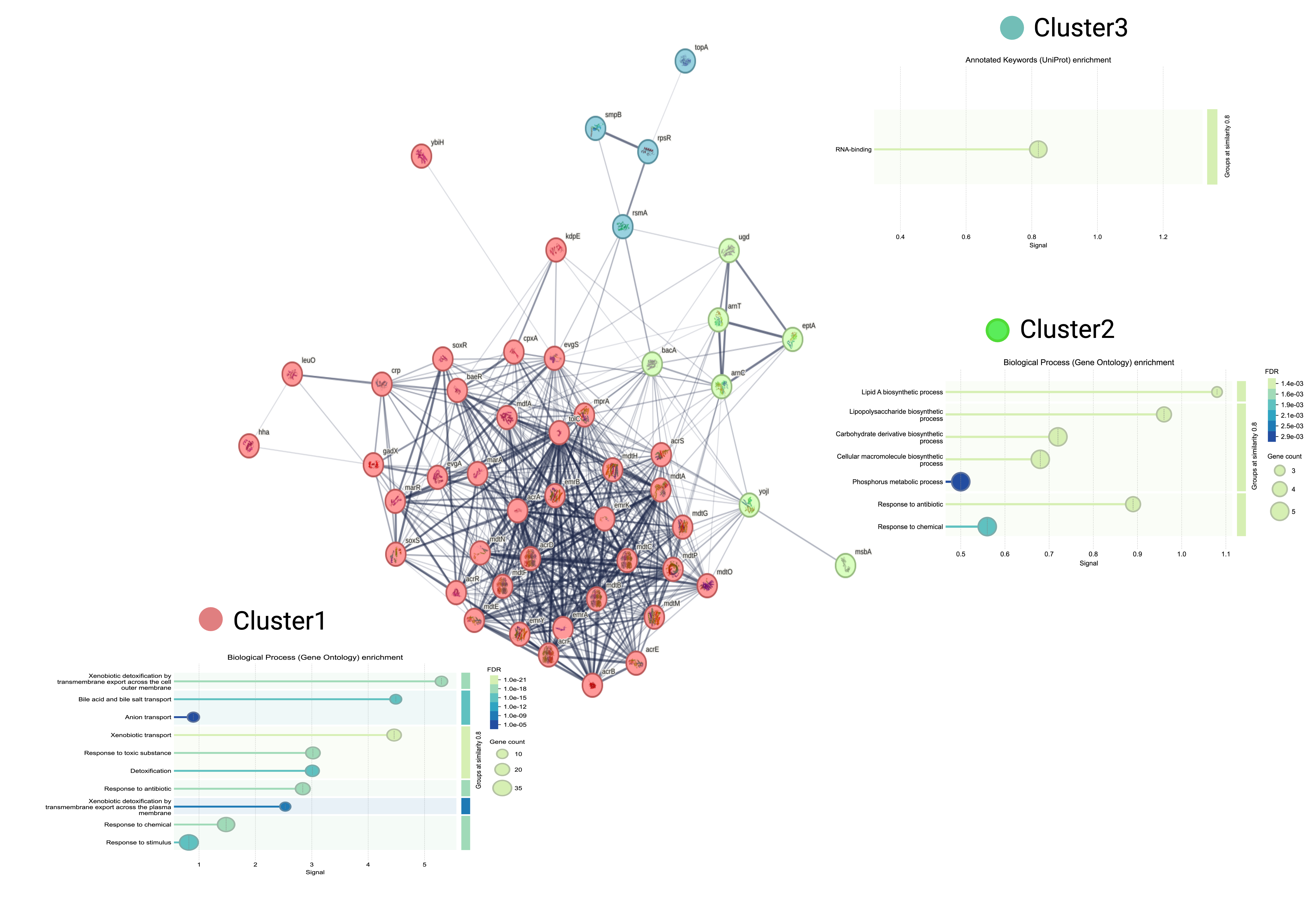

Supplement: Supplementary file 5 [file Image_1.tiff]
